# Simple model of saturable localised surface plasmon

**DOI:** 10.1038/s41598-018-20880-6

**Published:** 2018-02-08

**Authors:** Hisaki Oka, Yasuo Ohdaira

**Affiliations:** Faculty of engineering, Niigata university, 8050 Ikarashi nino-cho, Nishi-ku Niigata, 950-2102 Japan

## Abstract

Localised surface plasmons (LSPs) are now applied to various fields, such as bio-sensing, solar cell, molecular fluorescence enhancement and quantum-controlled devices at nanometre scale. Recent experiments show that LSPs are optically saturated by high-intensity light. Absorption saturation arises as a result of strong optical nonlinearity and cannot be explained by the conventional boson model of LSPs. Here, we propose a simple model of saturable LSPs using an effective dipole approximation. The strategy is to directly compare the classical linear optical response of an LSP with that obtained from a saturable quantum two-level system in the limit of weak excitation. The second quantization can then be performed by replacing a classical polarizability with a quantum dipole operator. Taking an ellipsoidal nanometal as an example, we analyse in detail the optical response of a single ellipsoidal nanometal to validate our model. Our numerical results show that the plasmon resonance frequency and spectral linewidth decrease as the aspect ratio of the ellipsoid increases, which is similar to the size dependence observed in early experiments.

## Introduction

Localised surface plasmons (LSPs) are quantized plasma oscillations formed near the surfaces of metal nanoparticles. In contrast to propagating surface plasmons, which often require carefully-constructed optical arrangement for phase matching, LSPs can be excited easily by direct irradiation of light. It is well known that LSPs can focus light to nanometre scale and strongly enhance the electric field near nanometals, which is called the antenna effect. The research field dealing with such plasmon characteristics is called “plasmonics”, and has been extensively investigated. In fact, many applications of LSPs, such as bio-sensing^1,2^, solar cell^3–5^ and molecular fluorescence enhancement^6–8^, have been reported.

For several years, the studies focused on the quantum nature of plasmons are now rapidly evolving, in which the light field interacts with matter at nanometre scale and plasmons play a role in controlling the light-matter interactions at the quantum level. This new research field is called “quantum plasmonics^9,10^”. Quantum plasmonics has opened up a new frontier in the study of the fundamental physics of LSPs^11–15^, namely vacuum Rabi splitting and the Fano effect, and now provide new potential applications of plasmons, such as single-photon sources^16,17^, entangled-photon sources^18,19^ and quantum-controlled devices at the nanometre scale^20–24^.

As is well known, however, the optical responses of LSPs are generally analysed using classical electromagnetism, in spite of the fact that LSPs are quantized plasma oscillations. This is because that the LSP antenna effect can be understood without the quantum nature of plasmon and can be explained simply by Maxwell’s equations. However, quantum plasmonics requires a quantum-mechanical treatment of plasmons, in other words, the second quantization of plasmons^25,26^. In particular, the understanding of the quantum properties of a single LSP, namely dipole moment and its relaxation rate, is required to understand the vacuum Rabi splitting in LSP-matter interaction, because the strong coupling is realized when the coupling constant rate *g* of LSP-matter interaction is lager than the relaxation rates of the LSP and the matter.

Conventionally, the second quantization of surface plasmons is based on the framework of cavity QED theory and is described by the boson model. In general, the boson model is justified by the fact that the Pauli exclusion principle hardly affects collective excitation. For example, in a lattice model of condensed-matter physics, the prohibition of two excitations at the same lattice corresponds directly to the Pauli exclusion principle. This prohibition component constitutes only *N*^−1^ of a collective excitation mode, where *N* is the number of lattice, and therefore can be ignored for $N\gg 1$. This boson approximation is valid for collective excitations of general quasiparticles. Recently, however, it has been experimentally reported that LSPs are optically saturated by high-intensity light^27^. Absorption saturation arises as a result of strong optical nonlinearity and cannot be explained by the above boson model. In contrast to propagating surface plasmons formed at the surface of planar metallic films, LSPs are localised in literally at nanometre scale. Therefore, the Pauli exclusion principle might be non-negligible, especially for small metal nanoparticles.

In this study, we propose a simple model of a saturable LSP for small metal nanoparticles using an effective dipole approximation. Our strategy is to directly compare the classical linear optical response of an LSP with that obtained from a saturable quantum two-level system. The second quantization is then performed by replacing a classical polarizability of the LSP with a quantum dipole operator described by the two-level system. Taking an ellipsoidal nanometal as an example, we introduce an optical response function of an LSP, based on the optical Bloch equations, and validate our method by analysing in detail the size dependence of the plasmon resonance frequency and the relaxation decay rate of a single LSP. Our numerical results show that the plasmon resonance frequency and spectral linewidth decrease as the aspect ratio of the ellipsoidal nanometal increases, which is similar to the size dependence observed in early experiments.

## Results

### Second quantization of LSPs using effective dipole approximation

**Figure 1:**
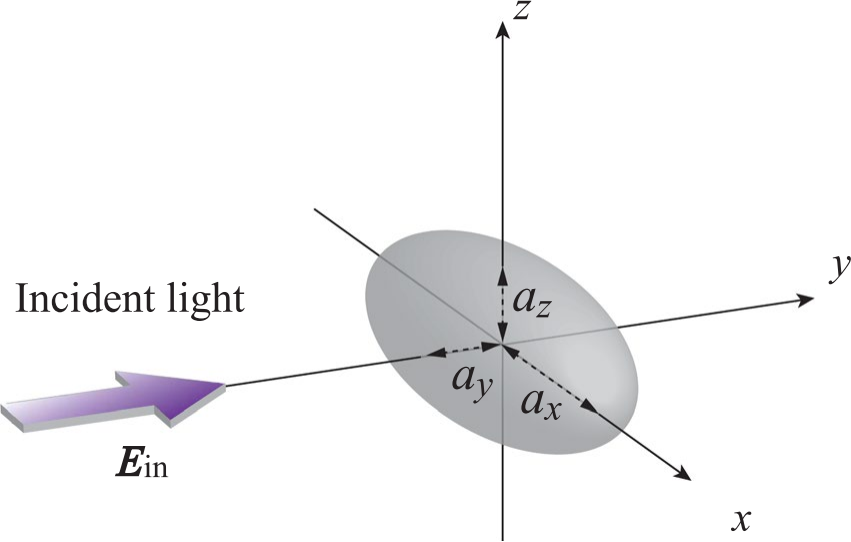
Schematic of analytical model. An ellipsoidal nanometal with semiaxes *a*_*x*_, *a*_*y*_ and *a*_*z*_ is located at the origin.

We start by considering a small metal ellipsoid with semiaxes *a*_*x*_, *a*_*y*_ and *a*_*z*_ interacting with incident light ***E***_in_ with a wavelength of *λ*, as depicted in Fig. 1. Assuming $a\ll \lambda$, we restrict ourselves to dipole radiation from the metal ellipsoid, because the quadrupole and magnetic dipole radiations are both negligibly small. When we focus on the quasi-static approximation^28^ and plasmon excitation at low-intensity light, dipole moment ***p***_*i*_ along the principal axes (*i* = *x*, *y*, *z*) can be described as a linear response of *E*_in,*i*_, given by

1 $${p}_{i}={\varepsilon }_{0}{\varepsilon }_{m}{\alpha }_{i}{E}_{{\rm{in}},i},$$

where *ε*_*m*_ is the dielectric constant of the background. The polarizability along the principal axes, *α*_*i*_, depends on the complex dielectric function *ε*(*ω*) of the ellipsoid:

2 $${\alpha }_{i}=\frac{4\pi {a}_{x}{a}_{y}{a}_{z}}{3}\frac{\varepsilon (\omega )-{\varepsilon }_{m}}{{\varepsilon }_{m}+{L}_{i}(\varepsilon (\omega )-{\varepsilon }_{m})}.$$

*L*_*i*_ is a geometrical factor given by

3 $${L}_{i}=\frac{{a}_{x}{a}_{y}{a}_{z}}{2}{\int }_{0}^{\infty }\frac{dq}{({a}_{i}^{2}+q)f(q)},$$

where $$f(q)={\{(q+{a}_{x}^{2})(q+{a}_{y}^{2})(q+{a}_{z}^{2})\}}^{\mathrm{1/2}}$$ and ∑_*i*_*L*_*i*_ = 1.

In order to quantize the LSP of the ellipsoidal nanometal, we follow the experimental facts^29^: The LSP absorbs a photon with a specific energy *ћω*_*R*_ and the LSP spectrum can be approximated by Lorentzian function. First, we derive the Lorentzian function from Eq. (2). By assuming the incident light with a frequency close to *ω*_*R*_, we approximate Eq. (2) by taking the Taylor expansion of *ε*(*ω*) around *ω*_*R*_. The plasmon resonance enhancement takes place under the condition that |*ε*_*m*_ + *L*_*i*_(*ε*(*ω*) − *ε*_*m*_)| is a minimum, which for the case of small or slowly-varying Im[*ε*(*ω*)] around *ω*_*R*_ can simplify to

4 $${\rm{Re}}[\varepsilon ({\omega }_{R})]=\frac{{\varepsilon }_{m}({L}_{i}-\mathrm{1)}}{{L}_{i}}.$$

Using this condition, the complex dielectric function *ε*(*ω*) can be approximated as^14^

5 $$\varepsilon (\omega )\approx \frac{{\varepsilon }_{m}({L}_{i}-1)}{{L}_{i}}+\frac{d{\rm{R}}{\rm{e}}[\varepsilon (\omega )]}{d\omega }{|}_{\omega ={\omega }_{R}}(\omega -{\omega }_{R})+i{\rm{I}}{\rm{m}}[{\omega }_{R}],$$

where we assume *d*Im[*ε*(*ω*_*R*_)]/*dω* ≈ 0. By substituting Eq. (5) into Eq. (2), we obtain

6 $${\alpha }_{i}\approx \frac{4\pi {a}_{x}{a}_{y}{a}_{z}}{3{L}_{i}}(1+\frac{{\varepsilon }_{m}}{\eta {L}_{i}}\frac{1}{{\omega }_{R}-\omega -i\gamma }),$$

where

7 $$\eta =\frac{d{\rm{R}}{\rm{e}}[\varepsilon (\omega )]}{d\omega }{|}_{\omega ={\omega }_{R}}$$

and

8 $$\gamma ={\rm{Im}}[\varepsilon ({\omega }_{R})]{\eta }^{-1}.$$

The factor $|{\varepsilon }_{m}{\eta }^{-1}{L}_{i}^{-1}|$ in Eq. (6) yields the enhancement of the electric field by plasmon resonance. For the case of large plasmon enhancement, $|{\varepsilon }_{m}{\eta }^{-1}{L}_{i}^{-1}|\gg 1$, the first term in parenthesis in Eq. (6) can be ignored, and we can rewrite Eq. (1) as the shape of the Lorentzian function,

9 $${p}_{i}=\frac{4\pi {\varepsilon }_{0}{\varepsilon }_{m}^{2}{a}_{x}{a}_{y}{a}_{z}}{3\eta {L}_{i}^{2}}\frac{1}{{\omega }_{R}-\omega -i\gamma }{E}_{{\rm{in}},i}.$$

Next, we follow another experimental fact that the LSPs are saturable^27^, and derive the dipole moment quantum-mechanically by considering a saturable quantum dipole operator. The simplest way to treat the saturation is to adopt the dipole operator of a two-level system, defined as ${\hat{p}}_{i}={d}_{i}(|e\rangle \langle g|+|g\rangle \langle e|)$, where |*g*〉 and |*e*〉 are the ground and excited states of a quantum two-level system and *d*_*i*_ is the dipole moment along the *i* axis. In the limit of weak excitation, the optical response of arbitrary quantum two-level systems can be approximated by

10 $$\langle {\hat{p}}_{i}\rangle =\frac{{|{d}_{i}|}^{2}}{\hslash }\frac{1}{{\omega }_{0}-\omega -i{\rm{\Gamma }}}{E}_{{\rm{i}}{\rm{n}},i},$$

where Γ is the dipole relaxation rate and *ω*_0_ is the energy difference between |*g*〉 and |*e*〉.

**Figure 2:**
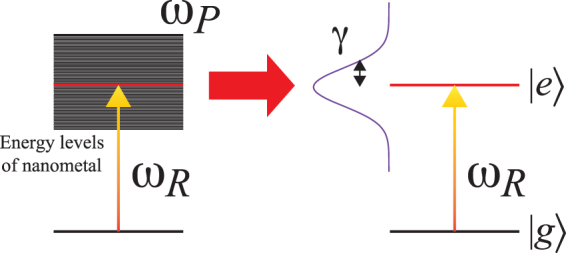
Schematic of the effective dipole approximation of LSP, where *ω*_*P*_ is the plasma frequency and the red line indicates the plasmon resonance, *ω*_*R*_.

Finally, we compare the classical *p*_*i*_ of Eq. (9) with the quantum-mechanical $\langle {\hat{p}}_{i}\rangle$ of Eq. (10). When the relations

11 $$\frac{4\pi {\varepsilon }_{0}{\varepsilon }_{m}^{2}{a}_{x}{a}_{y}{a}_{z}}{3\eta {L}_{i}^{2}}=\frac{{|{d}_{i}|}^{2}}{\hslash },$$

12 $${\omega }_{R}={\omega }_{0},$$

and

13 $$\gamma ={\rm{\Gamma }},$$

are satisfied, equation (9) becomes the same as Eq. (10). The LSP of the ellipsoidal nanometal can thus be reduced to an effective quantum two-level system with plasmon resonance frequency *ω*_*R*_ and dipole relaxation rate *γ*, as depicted in Fig. 2. The Hamiltonian of the LSP is given by $\hat{H}=\hslash {\omega }_{R}|e\rangle \langle e|$.

It should be noted that, for the case of LSP excitation by low-intensity light, the nonlinearity of the LSP is negligibly small. In this case, the LSP can be approximated by using a bosonic operator $\hat{c}$ instead of ${\hat{p}}_{i}$ in Eq. (10), as can be seen in the conventional quantization methods of surface plasmons.

### Plasmonic Bloch equations

Here, we extend $\langle {\hat{p}}_{i}\rangle$ to the case for no limit of weak excitation, presuming that the light polarization is parallel to a semiaxis *a*_*i*_ of the ellipsoid. In general, fully quantum-mechanical dynamics of a two-level system can be described by the quantum Langevin equations. Introducing the dipole operator ${\hat{\sigma }}_{-}=|g\rangle \langle e|$ and the inversion operator ${\hat{\sigma }}_{z}={2}^{-1}(|e\rangle \langle e|-|g\rangle \langle g|)$, the quantum Langevin equations are given by

14 $$\frac{d}{dt}{\hat{\sigma }}_{-}=-(\gamma +i{\omega }_{R}){\hat{\sigma }}_{-}+\sqrt{2\gamma }{\hat{\sigma }}_{z}{\hat{b}}_{{\rm{in}}}(t)$$

and

15 $$\frac{d}{dt}{\hat{\sigma }}_{z}=-2\gamma ({\hat{\sigma }}_{z}+\frac{1}{2})-2\sqrt{2\gamma }\{{\hat{\sigma }}_{+}{\hat{b}}_{{\rm{i}}{\rm{n}}}(t)+{\hat{b}}_{{\rm{i}}{\rm{n}}}^{\dagger }(t){\hat{\sigma }}_{-}\},$$

where ${\hat{\sigma }}_{+}={\hat{\sigma }}_{-}^{\dagger }$ and ${\hat{b}}_{{\rm{in}}}(t)$ is an input filed describing quantum fluctuation. With the input-output relation, ${\hat{b}}_{{\rm{in}}}(t)+{\hat{b}}_{{\rm{out}}}(t)=\sqrt{2\gamma }{\hat{\sigma }}_{-}$, Eqs (14) and (15) can be directly applied to the analysis of the quantum properties of photons emitted from a single LSP, such as single- photon and entangled-photon generations.

In this study, however, we focus on the optical response of LSPs driven by classical light in order to compare to conventional experimental results. In this case, the quantum Langevin equations can be reduced to the optical Bloch equations, by calculating the expectation values of operators, described as

16 $$\frac{d}{dt}\langle {\hat{\sigma }}_{-}\rangle =-(\gamma +i{\omega }_{R})\langle {\hat{\sigma }}_{-}\rangle -i\frac{2{d}_{i}E(t)}{\hslash }\langle {\hat{\sigma }}_{z}\rangle $$

and

17 $$\frac{d}{dt}\langle {\hat{\sigma }}_{z}\rangle =-2\gamma (\langle {\hat{\sigma }}_{z}\rangle +\frac{1}{2})-i\frac{{d}_{i}}{\hslash }\{\langle {\hat{\sigma }}_{-}\rangle {E}^{\ast }(t)-{\langle {\hat{\sigma }}_{-}\rangle }^{\ast }E(t)\}.$$

As in the second quantization of the LSP, we consider a cw incident light given by *E*(*t*) = *E*_in,*i*_ exp(−*iωt*). In the case of the cw incident light, it is possible to describe the response of the LSP in terms of the steady state dipole expectation value ${\langle {\hat{\sigma }}_{-}\rangle }_{s}$ oscillating at the same frequency as *E*(*t*). $\langle {\hat{\sigma }}_{z}\rangle$ then oscillates slowly as compared to the frequency *ω*, and hence ${\langle {\hat{\sigma }}_{z}\rangle }_{s}$ can be considered as a constant. The steady state dipole can then be obtained from Eq. (16) as

18 $${\langle {\hat{\sigma }}_{-}\rangle }_{s}=-\frac{2{d}_{i}{E}_{{\rm{in}},i}}{\hslash }\frac{1}{{\omega }_{R}-\omega -i\gamma }{\langle {\hat{\sigma }}_{z}\rangle }_{s}.$$

Equation (18) shows that ${\langle {\hat{\sigma }}_{-}\rangle }_{s}$ is proportional to the steady state value of the inversion ${\langle {\hat{\sigma }}_{z}\rangle }_{s}$. This steady state value can be obtained from Eq. (17), using the relation between dipole and inversion given by Eq. (18), and is given by

19 $${\langle {\hat{\sigma }}_{z}\rangle }_{s}=-\frac{{({\omega }_{R}-\omega )}^{2}+{\gamma }^{2}}{\mathrm{2\{(}{\omega }_{R}-\omega {)}^{2}+{\gamma }^{2}\}+{{\rm{\Omega }}}^{2}},$$

where Ω = 2*d*_*i*_*E*_*in*,*i*_*ћ*^−1^ is the Rabi frequency. Using Eq. (19), ${\langle {\hat{\sigma }}_{-}\rangle }_{s}$ can be rewritten as

20 $${\langle {\hat{\sigma }}_{-}\rangle }_{s}=\frac{2{d}_{i}}{\hslash }\frac{{\omega }_{R}-\omega +i\gamma }{\mathrm{2\{(}{\omega }_{R}-\omega {)}^{2}+{\gamma }^{2}\}+{{\rm{\Omega }}}^{2}}{E}_{{\rm{in}},i}\mathrm{.}$$

Since $\langle {\hat{p}}_{i}\rangle ={d}_{i}{\langle \sigma \rangle }_{-}$, we finally obtain $\langle {\hat{p}}_{i}\rangle$ as

21 $$\langle {\hat{p}}_{i}\rangle =\frac{4\pi {\varepsilon }_{0}{\varepsilon }_{m}^{2}{a}_{x}{a}_{y}{a}_{z}}{3\eta {L}_{i}^{2}}\frac{\mathrm{2(}{\omega }_{R}-\omega +i\gamma )}{\mathrm{2\{(}{\omega }_{R}-\omega {)}^{2}+{\gamma }^{2}\}+{{\rm{\Omega }}}^{2}}{E}_{{\rm{in}},i},$$

where Eq. (11) is used. This equation describes the saturation of LSP in terms of the dependence of the inversion ${\langle {\hat{\sigma }}_{z}\rangle }_{s}$ on the intensity of the incident light. In the limit of high-intensity incident light (Ω^2^ → ∞), the LSP is completely saturated and the inversion is ${\langle {\hat{\sigma }}_{z}\rangle }_{s}=0$, leading to ${\langle {\hat{\sigma }}_{-}\rangle }_{s}=\langle {\hat{p}}_{i}\rangle =0$. In the limit of weak excitation (Ω^2^ → 0), the LSP is in the ground state, i.e., ${\langle {\hat{\sigma }}_{z}\rangle }_{s}=-\frac{1}{2}$, and $\langle {\hat{p}}_{i}\rangle$ is the same as in Eq. (10).

Thus, by introducing the optical Bloch equations for LSPs, our quantization method can be applied to any intensity of incident light. Hereinafter, in this work, we refer to the above Bloch equations as plasmonic Bloch equations.

### Optical response function of a single LSP

In order to compare the optical response of the plasmonic Bloch equations to the experimental results, we here introduce an optical response function *χ* of a single quantized LSP. The imaginary part of *χ* corresponds to the spectrum of a single LSP. From Eq. (21), *χ* is simply defined as

22 $$\langle {\hat{p}}_{i}\rangle ={\varepsilon }_{m}{\varepsilon }_{0}\chi ({{\rm{\Omega }}}^{2}){E}_{{\rm{in}},i}$$

with

23 $$\chi ({{\rm{\Omega }}}^{2})=\frac{4\pi {\varepsilon }_{m}{a}_{x}{a}_{y}{a}_{z}}{3\eta {L}_{i}^{2}}\frac{\mathrm{2(}{\omega }_{R}-\omega +i\gamma )}{\mathrm{2\{(}{\omega }_{R}-\omega {)}^{2}+{\gamma }^{2}\}+{{\rm{\Omega }}}^{2}}\mathrm{.}$$

Though *ω*_*R*_ and *η* are formally derived from Eqs (4) and (7), respectively, their values cannot be determined without an explicit expression of *ε*(*ω*). In what follows, for simplicity, we consider a noble nanometal and use the Drude model, $\varepsilon (\omega )={\varepsilon }_{\infty }-{\omega }_{P}^{2}{({\omega }^{2}+i2\omega \gamma )}^{-1}$, where *ω*_*P*_ is the plasma frequency and *ε*_∞_ is the dielectric constant of the nanometal. From Eq. (4), *ω*_*R*_ can be derived as

24 $${\omega }_{R}\approx \frac{{\omega }_{P}}{{\{{\varepsilon }_{\infty }+{\varepsilon }_{m}({L}_{i}^{-1}-\mathrm{1)\}}}^{\mathrm{1/2}}},$$

where we presume ${\omega }_{P}^{2}\gg {\gamma }^{2}$. Similarly *η* can read

25 $$\eta =\frac{2{\omega }_{P}^{2}{\omega }_{R}}{{(4{\gamma }^{2}+{\omega }_{R}^{2})}^{2}}\approx \frac{2{\omega }_{P}^{2}}{{\omega }_{R}^{3}}.$$

The remaining unknown parameter is *γ*, however it cannot be derived directly from our method. Since *γ* corresponds to radiative relaxation of a single LSP, we evaluate it from the spontaneous emission rate, given by

26 $$2\gamma =\frac{{\omega }_{R}^{3}{|{d}_{i}|}^{2}}{\pi {\varepsilon }_{m}{\varepsilon }_{0}\hslash {c}^{3}}.$$

Using Eqs (11) and (25), |*d*_*i*_|^2^ can be rewritten as

27 $${|{d}_{i}|}^{2}\approx \frac{4\pi {\varepsilon }_{0}{\varepsilon }_{m}^{2}{a}_{x}{a}_{y}{a}_{z}\hslash {\omega }_{R}^{3}}{6{\omega }_{P}^{2}{L}_{i}^{2}}.$$

By substituting Eq. (27) into Eq. (26) and using Eq. (24), we obtain

28 $$2\gamma \approx \frac{2{\varepsilon }_{m}{a}_{x}{a}_{y}{a}_{z}{\omega }_{P}^{4}}{3{c}^{3}{L}_{i}^{2}{\{{\varepsilon }_{{\rm{\infty }}}+{\varepsilon }_{m}({L}_{i}^{-1}-1)\}}^{3}}.$$

Thus, the plasmon resonance frequency *ω*_*R*_, plasmon radiative decay rate *γ*, and optical response function *χ* for a single LSP can be expressed in terms of only the plasmon and geometrical parameters of nanometals, namely, *ω*_*P*_, *a*_*i*_, *L*_*i*_, *ε*_*m*_ and *ε*_∞_.

### Optical spectra of a single LSP: Dependence on metal particle size

**Figure 3:**
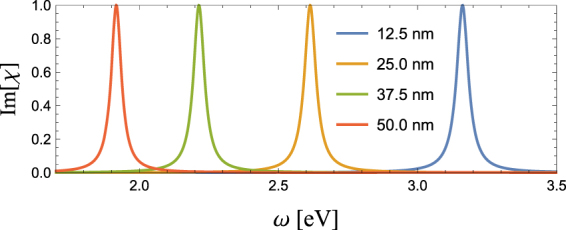
Im[*χ*] as a function of *ω* for *a*_*x*_ = 12.5, 25.0, 37.5 and 50.0 nm.

**Figure 4:**
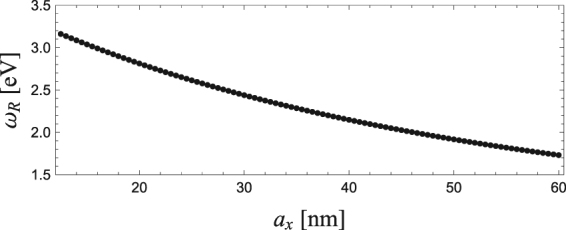
*ω*_*R*_ as a function of *a*_*x*_.

**Figure 5:**
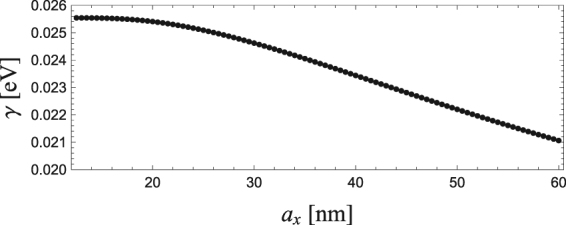
*γ* as a function of *a*_*x*_.

Here, we numerically analyse the particle-size dependence of optical spectra obtained from a single LSP using Im[*χ*]. The details of calculation parameters are given in the section of Methods. Figure 3 shows Im[*χ*] for *a*_*x*_ = 12.5, 25.0, 37.5 and 50.0 nm, normalized by its peak maximum at *ω*_*R*_. Both the central frequency *ω*_*R*_ and FWHM 2*γ* decrease as *a*_*x*_ increases. Details of the change in *ω*_*R*_ and *γ* are shown in Figs 4 and 5, respectively, as a function of *a*_*x*_. One can find that *ω*_*R*_ decreases monotonically as *a*_*x*_ increases, whereas *γ* decreases gradually, in particular in the range of small aspect ratio *a*_*x*_/*a* < 2: From *a*_*x*_ = 12.5 to 30 nm *ω*_*R*_ decreases from 3.2 to 2.5 eV, whereas *γ* is nearly unchanged from 25.5 to 25 meV.

**Figure 6:**
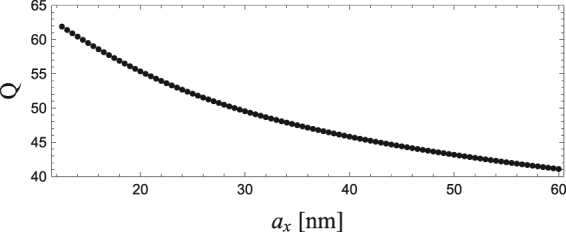
Q factor as a function of *a*_*x*_.

A similar tendency for *γ* to decrease as the aspect ratio increases can be found experimentally for gold nanorods^30^. For comparison with the experiment, the quality factors *Q* = *ω*_*R*_/2*γ* are plotted in Fig. 6. The Q values observed in the experiments are, however, smaller, being around a third of that obtained from our method. In addition, the size dependence exhibits the opposite trend: *Q* obtained from our method gradually decreases with increase in *a*_*x*_, whereas *Q* in the experiment increases with increase in *a*_*x*_ and saturates for large *a*_*x*_. This is due to the inhomogeneity of the nanometals in the actual experiment. Our result for size dependency of *ω*_*R*_ indicates that, for small *a*_*x*_, the small difference in *a*_*x*_, Δ*a*_*x*_, causes large inhomogeneous linewidth: For example, for *a*_*x*_ < 20 nm, a size inhomogeneity of Δ*a* = 0.5 nm leads to an energy fluctuation of Δ*ω*_*R*_ ≈ 25 meV, comparable to *γ*. Therefore, for small *a*_*x*_, size inhomogeneities in the nanometals directly lead to overestimation of the FWHM of the spectra. On the other hand, for large *a*_*x*_ > 50 nm, errors of Δ*a* = 0.5 nm lead to Δ*ω*_*R*_ < 10 meV, which is smaller than *γ*, and have little influence on the FWHM. Consequently, in the actual experiment, *Q* values for small *a*_*x*_ can become smaller than theoretically predicted values owing to size inhomogeneity of nanometals, and exhibit a tendency to increase as *a*_*x*_ increases. The size dependence of *Q* observed in the experiment can thus be explained qualitatively using *ω*_*R*_ and *γ* for a single LSP, which can be obtained from our method.

## Summary and Discussion

In summary, we have proposed a simple model of a saturable LSP using an effective dipole approximation. Taking an ellipsoidal nanometal as an example, we also have derived an optical response function of a single LSP from plasmonic Bloch equations, based on the optical Bloch equations, and have analysed in detail the size dependence of *ω*_*R*_ and *γ* of a single LSP. We have shown that *ω*_*R*_ and *γ* decrease as the aspect ratio of an ellipsoid increases, which is similar to the size dependence observed in the early experiments. We also have shown that, for small nanometals, *ω*_*R*_ is very sensitive to the inhomogeneity of particle size, leading to broadening of the FWHM of spectra obtained from a single LSP.

Although in this study we adopt a simple two-level model to describe the saturation of LSPs, a more rigorous approach, e.g., using lattice model for finite systems, could also be developed. However, we would like to emphasize that the proposed model can be simply applied to various shapes of LSP by varying the semiaxes of *a*_*x*_, *a*_*y*_ and *a*_*z*_, for example ${a}_{x}={a}_{y}\gg {a}_{z}$ for a disk-like nanometal or ${a}_{x}\gg {a}_{y}={a}_{z}$ for a rod-like nanometal, without explicitly considering the system size *N* of lattice model. In fact, the optical response of a single LSP is sensitive to the shape of nanometal, and hence our ellipsoidal model is quite useful. In addition, if we use the Drude-Lorentz model for the complex dielectric function *ε*(*ω*), the range of application of our method can be easily extended. Thus, our proposed method has advantages in simplicity and usability.

Finally, we should refer to the saturation of *ω*_*R*_ for large *a*, strictly speaking, less dependence of *ω*_*R*_ on *a*. This is because that *ω*_*R*_ approaches to the bulk limit, in which boson model is approximately valid. In fact, for large semiaxis *a*_*x*_, *ω*_*R*_ becomes almost constant. However, in this study, we presume a small nanometal to analyse the saturation effect of LSP and ignore higher-order multipole radiations. Therefore, if we would like to analyse the connection to the bulk limit rigorously, we have to consider the Mie scattering theory, which is beyond our model. Since the lowest-order approximation of scattering problem, used in this study, is adequate for nanoparticles of dimensions below 100 nm^28^, our proposed method is applicable for small nanoparticles. We hope that our results will facilitate the spread of the research field of quantum plasmonics.

## Methods

### Plasmonic parameters

In the calculation of optical spectra of a single LSP, we use the plasmon parameters of *ω*_*P*_ = 11.586 eV and *ε*_∞_ = 8.926^31^. For the background and semiaxes of the ellipsoidal nanometal we refer to ref.^30^: *ε*_*m*_ = 2.25, *a*_*y*_ = *a*_*z*_ = *a* = 12.5 nm (fixed) and *a*_*x*_ varies from 12.5 to 60 nm.

### Randomly oriented dipoles

|*d*_*i*_| and *γ* in the main text are obtained for a single LSP oriented in the same direction as the polarization of incident light. In realistic systems, however, the LSPs might be randomly oriented. In order to more accurately evaluate *d*_*i*_ and *γ* for comparison with the ensemble average of experimental results, we have to replace |*d*_*i*_|^2^ → ∑_*i*_|*d*_*i*_|^2^3^−1^ in Eq. (26). In the actual calculation, randomly oriented dipoles are adopted.
